# Genetic Variation in Selenoprotein Genes, Lifestyle, and Risk of Colon and Rectal Cancer

**DOI:** 10.1371/journal.pone.0037312

**Published:** 2012-05-17

**Authors:** Martha L. Slattery, Abbie Lundgreen, Bill Welbourn, Christopher Corcoran, Roger K. Wolff

**Affiliations:** 1 Department of Internal Medicine, University of Utah Health Sciences Center, Salt Lake City, Utah, United States of America; 2 Department of Mathematics and Statistics, Utah State University, Logan, Utah, United States of America; University of Aberdeen, United Kingdom

## Abstract

**Background:**

Associations between selenium and cancer have directed attention to role of selenoproteins in the carcinogenic process.

**Methods:**

We used data from two population-based case-control studies of colon (n = 1555 cases, 1956 controls) and rectal (n = 754 cases, 959 controls) cancer. We evaluated the association between genetic variation in *TXNRD1*, *TXNRD2*, *TXNRD3*, *C11orf31 (SelH)*, *SelW*, *SelN1*, *SelS*, *SepX*, and *SeP15* with colorectal cancer risk.

**Results:**

After adjustment for multiple comparisons, several associations were observed. Two SNPs in *TXNRD3* were associated with rectal cancer (rs11718498 dominant OR 1.42 95% CI 1.16,1.74 pACT 0.0036 and rs9637365 recessive 0.70 95% CI 0.55,0.90 pACT 0.0208). Four SNPs in *SepN1* were associated with rectal cancer (rs11247735 recessive OR 1.30 95% CI 1.04,1.63 pACT 0.0410; rs2072749 GGvsAA OR 0.53 95% CI 0.36,0.80 pACT 0.0159; rs4659382 recessive OR 0.58 95% CI 0.39,0.86 pACT 0.0247; rs718391 dominant OR 0.76 95% CI 0.62,0.94 pACT 0.0300). Interaction between these genes and exposures that could influence these genes showed numerous significant associations after adjustment for multiple comparisons. Two SNPs in *TXNRD1* and four SNPs in *TXNRD2* interacted with aspirin/NSAID to influence colon cancer; one SNP in *TXNRD1*, two SNPs in *TXNRD2*, and one SNP in *TXNRD3* interacted with aspirin/NSAIDs to influence rectal cancer. Five SNPs in *TXNRD2* and one in *SelS*, *SeP15*, and *SelW1* interacted with estrogen to modify colon cancer risk; one SNP in *SelW1* interacted with estrogen to alter rectal cancer risk. Several SNPs in this candidate pathway influenced survival after diagnosis with colon cancer (*SeP15* and *SepX1 increased HRR*) and rectal cancer (*SepX1 increased HRR*).

**Conclusions:**

Findings support an association between selenoprotein genes and colon and rectal cancer development and survival after diagnosis. Given the interactions observed, it is likely that the impact of cancer susceptibility from genotype is modified by lifestyle.

## Introduction

Selenoproteins are a class of proteins with the amino acid selenocysteine that contains the active form of selenium [1]. Studies reporting associations between selenium and cancer, and particularly colon cancer [2], [3], have directed attention to role of selenoproteins in the carcinogenic process. Twenty-five human selenoprotein genes have been identified [4], with most research focusing on the glutathione peroxidases (GPXs) and selenoprotein P (SePP1) which is involved in selenium transport [5]. However, given the biological properties of selenoproteins and their roles in control of intracellular redox environment, cellular growth, and defense against oxidative stress, it is feasible that other selenoproteins, such as thioredoxin reductase (TXNRD), selenoprotein W (SelW), selenoprotein N (SelN), selenoprotein S (SelS), selenoprotein H (SelH), selenoprotein X (SepX), and 15-kDa selenoprotein (SeP15) also may be involved in the carcinogenic process [4], [6].

Thioredoxin reductases catalyze the NADPH-dependent reduction of oxidized thioredoxin [7]. Thioredoxins are catalyzing agents that prevent cumulative oxidative stress, a factor that has been linked to cell death and carcinogenesis and is an important factor for controlling cellular redox regulation [8]. Humans have three thioredoxin reductases which reduce different substrates in different cellular compartments [9], [10], [11]: thioredoxin reductase 1 (TXNRD1), thioredoxin reductase 2 (TXNRD2), and thioredoxin reductase 3 (TXNRD3). SeP15 is structurally similar to the thioredoxin family. It is located primarily in the endoplasmic reticulum and is involved in the induction of apoptosis and exhibits redox activity [1], [12]. SepW has been shown to be expressed in the intestinal tract and studies have shown that it also exhibits oxidation-reduction activity and possible antioxidant properties [13], [14]. SelS attenuates inflammation by decreasing pro-inflammatory cytokines [15]. SelN, SelH and SelX, although thought to have biological functions that involve redox functions and antioxidant properties, have been less well studied [4], [14].

In this paper we evaluate associations between genetic polymorphism in *TXNRD1*, *TXNRD2*, *TXNRD3*, *C11orf31 (i.e. SelH)*, *SelW*, *SelN1*, *SelS*, *SepX*, and *SeP15* and colon and rectal cancer. Results on *GPX* and *SelP* from study data have been previously assessed [16]. Given the hypothesized association between these genes and oxidative stress, we evaluate diet and lifestyle exposures that may influence observed colorectal cancer risk associated with these genes. Dietary antioxidants have been associated with other genes that mediate oxidative stress [17] and could likewise interact with these genes. Cigarette smoking can increase levels of oxidative stress; use of aspirin and non-steroidal anti-inflammatory drugs can reduce inflammation and thus reduce oxidative stress; BMI has been associated with increased inflammation which can lead to oxidative stress. We evaluate estrogen status since studies have shown an association between estrogen status and selenium [18], [19]; HRT use has been shown to reduce risk of colorectal cancer. We also evaluate if genetic variation in these selenoprotein genes influences survival after diagnosis with colon or rectal cancer since previous studies shown that SeP15 is associated with metastasis of colon cancer cells [20]. This expands on the work of others that have proposed that a combination of low selenium and SNPs in selonoprotein genes can enhance the risk of colorectal cancer [14]

## Methods

Two study populations are included. The first, a population-based case-control study of colon cancer, included cases (n = 1,555) and controls (n = 1,956) identified between October 1, 1991 and September 30, 1994 living in the Twin Cities Metropolitan Area, Kaiser Permanente Medical Care Program of Northern California (KPMCP) and a seven-county area of Utah [21]. The second study used identical data collection methods as the first study but included population-based cases with cancer of the rectosigmoid junction or rectum (n = 754) and controls (n = 959) who were identified between May 1997 and May 2001 in Utah and KPMCP [22]. Eligible cases were between 30 and 79 years old at time of diagnosis, English speaking, mentally competent to complete the interview, no previous history of CRC, and no known (as indicated on the pathology report) familial adenomatous polyposis, ulcerative colitis, or Crohn's disease. Controls were matched to cases by sex and by 5-year age groups. At KPMCP, controls were randomly selected from membership lists. In Utah, controls 65 years and older were randomly selected from the Health Care Financing Administration lists and controls younger than 65 years were randomly selected from driver's license lists. In Minnesota, controls were selected from driver's license and state-identification lists. Study details have been reported [21], [22]. The Study was approved by the Institutional Review Board at the University of Utah. All participants signed informed consent.

Data were collected by trained and certified interviewers using laptop computers. All interviews were audio-taped and reviewed for quality control purposes [23]. The referent period for recall of diet and physical activity was two years prior to diagnosis for cases and prior to selection for controls. Detailed information was collected on diet [24], physical activity, medical history, cigarette smoking history, regular use of aspirin and non-steroidal anti-inflammatory drugs, and body size. Dietary data were collected on all participants using an extensive diet history questionnaire [25]. For those foods reported, we obtained information on quantity, frequency, and method of preparation. Foods were converted to nutrients using the Minnesota Nutrition Coding Center nutrient database. The body mass index (BMI) of kg/m^2^ was calculated from height measured at the time of the interview and weight recalled for the referent period of two years prior to diagnosis or selection. In instances where weight two years prior to diagnosis was unavailable, we used weight reported for five years prior to diagnosis or interview. Recalled weight was used to avoid possible misclassification of weight from weight loss attributed to cancer.

Tumor registry data were obtained to determine disease stage at diagnosis, months of survival after diagnosis, cause of death and contributing cause of death. Disease stage was categorized by Surveillance, Epidemiology, and End Results (SEER) staging of local, regional, and distant disease as well as by the American Joint Committee on Cancer (AJCC) staging criteria.

TagSNPs were selected using the following parameters: LD blocks were defined using a Caucasian LD map and an r^2^ = 0.8; minor allele frequency (MAF) >0.1; range = −1500 bps from the initiation codon to +1500 bps from the termination codon; and 1 SNP/LD bin. This procedure generated two markers for *SelS*, three for *SeP15*, five for *SelN1*, three for *SelW1*, two for *SepX1*, one for *C11of31*, eight for *TXNRD1*, twenty for *TXNRD2*, and five for *TXNRD3*. All markers were genotyped using a multiplexed bead-array assay format based on GoldenGate chemistry (Illumina, San Diego, California). A genotyping call rate of 99.85% was attained. Blinded internal replicates represented 4.4% of the sample set; the duplicate concordance rate was 100%. Individuals with missing genotype data were not included in the analysis for that specific marker.

Statistical analyses were performed for each study independently using SAS® version 9.2 (SAS Institute, Cary, NC). The minor allele frequency (MAF) and test for Hardy-Weinberg Equilibrium (HWE) were calculated among white controls using the SAS ALLELE procedure. We report odds ratios (ORs) and 95% confidence intervals (CIs) assessed from adjusted multiple logistic regression models adjusting for age, center, race/ethnicity, and sex, which were matching variables for the original studies. Analysis for interaction was based on tagSNPs within each gene. All other SNPs were evaluated first by comparing the heterozygote and homozygote variant to the homozygote wildtype and subsequently assessing the dominant and recessive models; the best fitting model is presented.

Diet and lifestyle variables for assessment with candidate genes were selected because of their biological plausibility for involvement in this candidate pathway. Interactions between genes and hypothesized exposures associated with inflammation and oxidative stress included daily consumption of vitamin C, vitamin E, selenium, and beta carotene, recent aspirin or NSAID use, cigarette smoking status, BMI, and estrogen status. Nutrients reported were categorized based on sex-specific quartiles among the controls, collapsing the second and third quartiles to form an intermediate group. In addition to the minimal adjustments, logistic regression models involving dietary variables also control for total energy intake. P values for interaction were determined using a 1df likelihood-ratio test comparing a full model that included an interaction term with a reduced model without an interaction term. For genetic and environmental factors that have a 20% prevalence among controls with have 80% power to detect an OR of 1.87 for colon cancer and 2.30 for rectal cancer; for those with a 30% prevalence we have power to detect a 1.77 for colon and 2.15 for rectal when using a 5% significance levels The p values based on 1 degree of freedom (1-df) Wald test statistics for the main effect models were adjusted for multiple comparisons taking into account tagSNPs within the gene, using the methods of Conneely and Boehnke [26] via R version 2.12.0 (R Foundation for Statistical Computing, Vienna, Austria). The interaction p values, based on 1-df likelihood-ratio tests, were adjusted using the step-down Bonferroni correction or the Holm's test [27]. Wald p values from the main effect models and interaction p values based on likelihood-ratio tests were used to calculate multiple comparisons. We consider a p value of 0.10 to be potentially important for adjusted main effects and survival analysis given the candidate pathway approach we have used in this study. Since we are using the highly conservative Bonferroni method for adjustment of multiple comparisons for interactions, we consider a p value of 0.15 or less as potentially important so that we are able to consider both type 1 and type 2 errors. Additionally, we used a maxT permutations procedure [28] to further evaluate interactions and correspondingly adjust for multiple comparisons. Using the highly efficient methods of Welbourn [29], 100,000 max T permutations were performed for GXE pairing. Hypothesis tests involving genotype and lifestyle exposure combinations between an individual SNP and a single lifestyle variable were mutually adjusted by comparing each observed test statistic to the permutation null distribution of the maximum test statistic over all tests conducted upon that SNP. This adjustment was then expanded to mutually adjust for all pairings between a single lifestyle variable and all SNPs within a gene. This method also allowed for partitioning of the data to better identify and categorize the most meaningful groups where the interactions occurred. The maxT statistic complements other methods of multiple comparison adjustment by further defining the interaction as well as by using a more robust permutations adjustment for multiple comparisons. For survival analysis, multiple comparison adjustments were done using the false discovery rate (FDR) adjusted p values using the SAS MULTTEST procedure.

Survival-months were calculated based on month and year of diagnosis and month and year of death or date of last contact. Associations between SNPs and risk of death due to colorectal cancer were evaluated using Cox proportional hazards models to obtain adjusted hazard rate ratios (HRRs) and corresponding 95% confidence intervals. We adjusted for age at diagnosis, study center, race, sex, tumor molecular phenotype, and AJCC stage to estimate HRRs and censored individuals at date of last contact or death. Tumor molecular phenotype was determined from DNA obtained from paraffin-embedded tissue. We have previously sequenced hot spots for *TP53 and* KRAS, and assessed CpG Island Methylator Phenotype (CIMP), and microsatellite instability (MSI) [30], [31], [32], [33].

## Results

The tagSNPs analyzed are shown in Table 1; all SNPs are in HWE. SNPs that were independently associated with colon and rectal cancer are shown in Table 2. Although three SNPs in *TXNRD1*, *TXNRD2* and *SelN1* were associated with colon cancer, none remained statistically significant after adjustment for multiple comparisons as indicated by the pACT. *TXNRD2* (3 SNPs), *TXNRD3* (3 SNPs), *SelN1* (3 SNPs), and *SepX1* (1 SNP) were associated with rectal cancer. While SNPs in *TXNRD2* and *SepX1* did not remain statistically significant after adjustment for multiple comparisons, those in *TXNRD3* and *SelN1* were statistically significant after multiple comparison adjustments with pACT.

**Table 1:** tagSNPs analyzed.

		Chromosome Location		Major/Minor Allele		FDR HWE
Symbol	Alias		SNP		MAF^1^	
*C11orf31*	*C17orf10*, *SELH*	11q12.1	rs9420	G/A	0.32	0.95
*SelS*	*AD-015*, *ADO15*	15q26.3	rs9874	A/G	0.14	1.00
	*MGC104346*, *MGC2553*		rs4965814	T/C	0.18	1.00
	*SBBI8*, *SEPS1*, *VIMP*					
*SeP15*		1p31	rs2783974	G/A	0.12	0.75
			rs486133	T/C	0.20	1.00
			rs9433110	G/A	0.07	0.95
*SelN1*	*FLJ24021*	1p36.13	rs718391	C/G	0.47	1.00
	*MDRS1*		rs2072749	A/G	0.27	1.00
	*RSMD1*		rs11247735	G/A	0.47	1.00
	*RSS*		rs4659382	C/G	0.28	0.96
	*SEPN*		rs2294228	T/G	0.21	1.00
*SelW1*	*SepW*	19q13.3	rs10412896	T/C	0.35	0.98
			rs3786777	G/T	0.49	1.00
			rs2042286	C/T	0.39	0.98
*SepX1*	*HSPC270*, *MGC3344*	16p13.3	rs13331553	T/C	0.29	1.00
	*MSRB1*, *SELR*, *SELX*		rs732510	A/G	0.43	1.00
*TXNRD1*	*GRIM-12*	12q23-q24.1	rs4964778	C/G	0.18	0.97
	*MGC9145*		rs4964779	T/C	0.11	1.00
	*TR*		rs4523760	T/C	0.23	0.74
	*TR1*		rs5018287	G/A	0.45	1.00
	*TRXR1*		rs4964287	C/T	0.32	0.91
	*TXNR*		rs17202060	C/T	0.34	0.58
			rs7962759	C/G	0.22	1.00
			rs11610799	G/C	0.08	1.00
*TXNRD2*	*SELZ*	22q11.21	rs1044732	A/G	0.15	0.95
	*TR*		rs3788305	A/G	0.47	1.00
	*TR-BETA*		rs3788306	T/C	0.30	1.00
	*TR3*		rs2073750	G/A	0.23	1.00
	*TRXR2*		rs9606173	A/T	0.15	0.96
			rs5992493	A/G	0.17	1.00
			rs3788314	G/A	0.46	1.00
			rs3788317	G/T	0.23	0.98
			rs7410379	G/A	0.29	1.00
			rs756661	T/C	0.45	0.97
			rs5748469	C/A	0.35	0.98
			rs17745445	G/A	0.15	1.00
			rs1978058	C/T	0.38	0.81
			rs8141691	G/A	0.37	0.68
			rs9306229	C/T	0.24	0.48
			rs4333017	C/T	0.14	0.98
			rs5746847	C/T	0.44	1.00
			rs9605030	C/T	0.14	1.00
			rs6518591	A/G	0.19	0.58
			rs2020917	C/T	0.27	0.97
*TXNRD3*	*TGR*	3q21.3	rs4679274	C/T	0.34	1.00
	*TR2*		rs777226	G/A	0.22	0.95
	*TRXR3*		rs777238	C/T	0.13	0.68
			rs9637365	C/T	0.42	0.85
			rs11718498	G/A	0.42	0.05

1 Minor Allele Frequency (MAF) and FDR-adjusted Hardy-Weinberg Equilibrium (FDR HWE) based on white control population.

**Table 2:** Associations between *TXNRD1*, *TXDRD2*, *TXNRD3*, *SelN1*, and *SepX1* and colon and rectal cancer.

Colon Cancer	Controls	Cases	OR^1^	(95% CI)		Raw *P*	*P*_ACT_
*TXNRD1* (rs17202060)						0.0209	0.1251
CC/CT	1722	1324	1.00				
TT	232	222	1.26	(1.04	1.54)		
*TXNRD2* (rs3788317)						0.0266	0.3341
GG/GT	1859	1448	1.00				
TT	96	107	1.38	(1.04	1.84)		
*SelN1* (rs4659382)							
CC/CG	1797	1458	1.00			0.0383	0.1428
GG	156	95	0.76	(0.58,	0.98)		
**Rectal Cancer**							
*TXNRD2* (rs1044732)						0.0361	0.4002
AA	685	575	1.00				
AG/GG	270	176	0.79	(0.63	0.98)		
*TXNRD2* (rs5748469)						0.0139	0.2017
CC/CA	833	620	1.00				
AA	125	134	1.40	(1.07	1.83)		
*TXNRD2* (rs5992493)						0.0277	0.3360
AA	619	521	1.00				
AG/GG	340	233	0.79	(0.65	0.98)		
*TXNRD3* (rs11718498)						0.0008	0.0036
GG	361	227	1.00				
GA/AA	598	527	1.42	(1.16	1.74)		
*TXNRD3* (rs4679274)						0.0339	0.0919
CC/CT	824	670	1.00				
TT	135	83	0.73	(0.54	0.98)		
*TXNRD3* (rs9637365)						0.0059	0.0208
CC/CT	757	631	1.00				
TT	202	123	0.70	(0.55	0.90)		
*SelN1 (rs11247735)*						0.0213	0.0410
GG/GA	753	554	1.00				
AA	206	200	1.30	(1.04,	1.63)		
*SelN1* (rs2072749)						0.0035	0.0159
AA	484	422	1.00				
AG	394	294	0.86	(0.70,	1.05)		
GG	81	38	0.53	(0.36,	0.80)		
*SelN1* (rs4659382)						0.0067	0.0247
CC/CG	876	716	1.00				
GG	81	38	0.58	(0.39,	0.86)		
*SelN1* (rs718391)						0.0113	0.0300
CC	250	239	1.00				
CG/GG	709	515	0.76	(0.62,	0.94)		
*SepX1* (rs732510)						0.0310	0.0565
AA/AG	763	563	1.00				
GG	192	187	1.29	(1.02,	1.63)		

1 Associations adjusted for age, sex, race, and study center.

We observed statistically significant interaction with aspirin/NSAIDs and smoking with several candidate genes (Table 3). The most common interaction with aspirin followed the pattern of lower risk for the variant allele among NSAID users. Interactions between aspirin/NSAIDs with *TXNRD1* rs4964778 remained statistically significant for colon cancer after adjustment for multiple comparison; rs17745445 of *TNXRD2* was borderline significant after adjustment for multiple comparison with the step-down Bonferroni correction. Two SNPs in *TXNRD2* interacted significant with cigarette smoking for colon cancer where those who smoked were at greater risk with the variant allele; associations were not statistically significant after adjustment for multiple comparisons. For rectal cancer four SNPs in *TXNRD1*, *TXNRD2*, and *TXNRD3* interacted with aspirin/NSAID use and two SNPs in *TXNRD1* interacted with cigarette smoking; the step-down Bonferroni correction was greater than 0.15 for all of these associationsFor rectal cancer and aspirin, the greatest effect of the genes appeared to be among non-NSAID users while among those who smoked cigarettes the variant allele appeared to reduce the risk of rectal cancer associated with smoking. The maxT, which is more robust for adjustment of multiple comparisons than the step-down Bonferroni correction, showed statistically significant interaction with all SNPS identified as interacting with aspirin/NSAID use for both colon and rectal cancer.

**Table 3:** Associations between *TXNRD* and selenoprotein SNPs, recent regular use of aspirin/NSAID, cigarette smoking and risk of colon and rectal cancer.

	Controls	Cases	OR^1^	(95% CI)		Controls	Cases	OR	(95% CI)		Wald *P*	Holm *P*	Interaction Level (L) Test^2^	
														maxT *P*
**Colon Cancer**	No Recent Aspirin/NSAID Use					Recent Aspirin/NSAID Use								
*TXNRD1* (rs4523760)											0.0234	0.1638	G = {1,2} & E = 1	<0.0001
TT	686	612	1			459	304	0.75	(0.62,	0.90)				
TC/CC	449	439	1.09	(0.92,	1.30)	345	180	0.59	(0.47,	0.72)				
*TXNRD1* (rs4964778)											0.0026	0.0208	G = {1,2} & E = 1	<0.0001
CC	779	691	1.00			524	350	0.76	(0.64,	0.90)				
CG/GG	356	361	1.14	(0.95,	1.37)	280	135	0.54	(0.43,	0.68)				
*TXNRD2* (rs17745445)											0.0039	0.0780	G = {1,2} & E = 1	0.0020
GG	855	756	1.00			580	375	0.74	(0.63,	0.87)				
GA/AA	281	297	1.20	(1.00,	1.46)	223	110	0.55	(0.43,	0.71)				
*TXNRD2* (rs3788314)											0.0198	0.3762	G = {1,2} & E = 0	<0.0001
GG	350	271	1.00			237	145	0.80	(0.62,	1.05)				
GA	561	527	1.22	(1.00,	1.48)	381	239	0.81	(0.65,	1.02)				
AA	225	251	1.41	(1.11,	1.80)	181	101	0.70	(0.52,	0.94)				
*TXNRD2* (rs5992493)											0.0207	0.3762	G = {1,2} & E = 0	0.0023
AA	794	691	1.00			553	349	0.73	(0.62,	0.87)				
AG/GG	342	362	1.20	(1.00,	1.43)	250	136	0.61	(0.48,	0.77)				
*TXNRD2* (rs756661)											0.0401	0.6817	G in {0 1) & E = 0	<0.0001
TT	353	364	1.00			257	141	0.53	(0.41,	0.68)				
TC	548	503	0.90	(0.75,	1.10)	373	239	0.63	(0.51,	0.79)				
CC	235	184	0.78	(0.61,	0.99)	172	105	0.62	(0.46,	0.82)				
	Non-Smoker/Non-Recent Smoker					Recent Smoker								
*TXNRD2* (rs17745445)											0.0388	0.7372	G = {1,2} & E = 1	0.4918
GG	1180	920	1.00			265	223	1.04	(0.85,	1.28)				
GA/AA	428	314	0.94	(0.79,	1.11)	81	95	1.47	(1.08,	2.00)				
*TXNRD2* (rs5992493)											0.0241	0.4820	G = {1,2} & E = 1	0.1540
AA	1102	846	1.00			254	206	1.02	(0.83,	1.26)				
AG/GG	506	388	0.97	(0.83,	1.14)	92	112	1.52	(1.13,	2.03)				
**Rectal Cancer**	No Recent Aspirin/NSAID Use					Recent Aspirin NSAID Use								
*TXNRD1* (rs4964778)											0.0380	0.3040	G = {1,2} & E = 1	0.0404
CC	364	321	1.00			283	198	0.80	(0.63,	1.02)				
CG/GG	157	156	1.15	(0.88,	1.50)	144	73	0.59	(0.43,	0.81)				
*TXNRD2* (rs1978058)											0.0446	0.8474	G = {0,1} & E = 1	0.0141
CC	203	214	1.00			190	110	0.56	(0.41,	0.75)				
CT	248	202	0.78	(0.60,	1.02)	186	119	0.62	(0.46,	0.84)				
TT	70	61	0.84	(0.57,	1.25)	51	42	0.80	(0.51,	1.26)				
*TXNRD2* (rs9606173)											0.0353	0.7060	G = 0 & E = 1	0.0145
AA	344	334	1.00			316	185	0.61	(0.48,	0.77)				
AT/TT	177	143	0.83	(0.63,	1.08)	111	86	0.80	(0.58,	1.10)				
*TXNRD3* (rs9637365)											0.0265	0.1325	G = {0,1} & E = 0	0.0002
CC	164	179	1.00			147	83	0.52	(0.37,	0.74)				
CT	241	226	0.86	(0.65,	1.14)	197	138	0.65	(0.48,	0.88)				
TT	116	72	0.55	(0.38,	0.79)	84	50	0.53	(0.35,	0.81)				
		Non-Smoker/Non-Recent Smoker				Recent Smoker								
*TXNRD1* (rs17202060)														
CC	369	237	1.00			64	76	1.82	(1.25,	2.64)	0.0274	0.2192	G = 0 & E = 0	0.1078
CT	329	290	1.38	(1.10,	1.73)	65	58	1.35	(0.91,	2.00)				
TT	110	75	1.06	(0.76,	1.48)	21	14	0.97	(0.48,	1.96)				

1 Odds Ratios (OR) and 95% Confidence Intervals (CI) adjusted for age, study center, race, and sex.

2 G = numerical coding (i.e., 0, 1, 2) for the SNP; E = numerical coding (i.e., 0, 1) for the environmental factor.

Only TXNRD3 rs11718498 and rs777226 were associated with vitamin E and beta carotene respectively after adjustment for multiple comparisons (Table S1) shows dietary variables associated with SNPs prior to adjustment and the corresponding p value after multiple comparison adjustment). In both instances those with low intake had reduced colon cancer risk in the presence of the variant genotype, while those with high intake were at reduced intake in the presence of wildtype and heterozygote variant.

We observed numerous statistically significant interactions between candidate genes, *TXNRD2*, *SelS*, *SeP15*, and *SelW1* and estrogen status for both colon and rectal cancer (Table 4). While the variant alleles often increased risk among those not exposed to estrogen, they appeared to reduce risk among those exposed to estrogen. Roughly 50% of the SNPs initially associated showed a significant interaction after adjustment for multiple comparisons. Utilization of the maxT highlighted the focus of the interactive effects with most interactions remained statistically significant with this approach. In general, the estrogen status had a more pronounced effect depending on genotype of these candidate selenoprotein genes.

**Table 4:** Associations between *TXNRD* and selenoprotein SNPs and estrogen and risk of colon and rectal cancer.

	Controls	Cases	OR^1^	(95% CI)		Controls	Cases	OR	(95% CI)		Wald *P*	Holm *P*	Interaction Level (L) Test^2^	
														maxT *P*
**Colon Cancer**	No Recent Estrogen Exposure					Recent Estrogen Exposure								
*TXNRD2* (rs17745445)											0.0011	0.0220	G = {1,2} & E = 1	0.0077
GG	410	336	1.00			251	180	0.72	(0.54,	0.95)				
GA/AA	113	113	1.24	(0.92,	1.68)	109	42	0.39	(0.26,	0.59)				
*TXNRD2* (rs3788314)											0.0015	0.0270	G = {1,2} & E = 1	0.0121
GG	177	121	1.00			88	73	1.01	(0.67,	1.52)				
GA	244	219	1.32	(0.98,	1.77)	186	102	0.65	(0.46,	0.94)				
AA	98	107	1.57	(1.09,	2.25)	86	46	0.62	(0.40,	0.98)				
*TXNRD2* (rs3788317)											0.0012	0.0228	G = {1,2} & E = 1	0.0083
GG	332	261	1.00			193	145	0.78	(0.58,	1.06)				
GT/TT	191	188	1.24	(0.96,	1.61)	167	77	0.47	(0.34,	0.67)				
*TXNRD2* (rs5992493)											0.0197	0.2955	G = {1,2} & E = 1	0.1093
AA	374	305	1.00			241	167	0.69	(0.52,	0.92)				
AG/GG	149	144	1.15	(0.87,	1.53)	119	55	0.46	(0.31,	0.67)				
*TXNRD2* (rs756661)														
TT	152	159	1.00			132	63	0.38	(0.25,	0.56)	0.0101	0.1717	G = 0 & E = 1	0.1076
TC	252	201	0.79	(0.59,	1.06)	163	112	0.55	(0.38,	0.79)				
CC	118	89	0.75	(0.52,	1.07)	65	46	0.58	(0.36,	0.92)				
*SelS* (rs9874)											0.0359	0.0718	G = {1,2} & E = 0	0.0109
AA	392	306	1.00			251	160	0.67	(0.50,	0.89)				
AG/GG	131	143	1.39	(1.05,	1.84)	110	62	0.56	(0.39,	0.82)				
*SeP15* (rs2783974)											0.0236	0.0708	G = 0 & E = 0	0.0018
GG	412	379	1.00			292	171	0.52	(0.40,	0.68)				
GA/AA	111	70	0.69	(0.50,	0.96)	69	51	0.66	(0.43,	1.01)				
*SepW1* (rs3786777)											0.0037	0.0111	G = 2 & E = 1	0.0168
GG/GT	399	320	1.00			259	178	0.7	(0.53,	0.93)				
TT	123	129	1.27	(0.95,	1.70)	102	44	0.43	(0.29,	0.64)				
**Rectal Cancer**														
*TXNRD2* (rs2073750)											0.0065	0.1300	G = 0 & E = 0	0.5276
GG	84	86	1.00			151	84	0.45	(0.29,	0.69)				
GA/AA	85	55	0.62	(0.39,	0.97)	98	77	0.64	(0.41,	1.01)				
*SepW1* (rs2042286)											0.0016	0.0048	G = 2 & E = 1	0.0423
CC/CT	151	119	1.00			211	150	0.77	(0.54,	1.09)				
TT	17	22	1.71	(0.87,	3.38)	38	10	0.28	(0.13,	0.59)				

1 Odds Ratios (OR) and 95% Confidence Intervals (CI) adjusted for age, study center, race, and sex.

2 G = numerical coding (i.e., 0, 1, 2) for the SNP; E = numerical coding (i.e., 0, 1) for the environmental factor.

*TXNRD1*, *TXNRD2*, *TXNRD3*, and *SelN1* interacted with BMI to alter risk of colon cancer and *TXNRD1* interacted with BMI to statistically alter risk associated with rectal cancer (Table 5). The adjusted risk for *SelN1* and colon cancer and both *TXNRD1* SNPs and rectal cancer remained statistically significant after adjustment for multiple comparisons. The pattern of association implied that the cancer risk associated with obesity was influenced by genotype.

**Table 5:** Interaction between *TXNRD* and selenoprotein SNPs and obesity and risk of colon cancer.

	Normal (<25)					Overweight (25–29)					Obese (> = 30)								
Colon Cancer	Controls	Cases	OR^1^	(95% CI)		Controls	Cases	OR	(95% CI)		Controls	Cases	OR	(95% CI)		Wald *P*	Holm *P*	Interaction Level Test^2^	maxT *P*
*TXNRD1* (rs4964779)																0.0375	0.3000	G = 0 & E = 2	<0.0001
TT	614	394	1.00			634	507	1.24	(1.04,	1.47)	307	341	1.70	(1.39,	2.08)				
TC/CC	144	110	1.19	(0.90,	1.57)	162	123	1.17	(0.90,	1.53)	91	75	1.27	(0.91,	1.77)				
*TXNRD2* (rs1044732)																0.0233	0.4660	G = {1,2} & E = 0	0.0928
AA	530	378	1.00			584	452	1.08	(0.90,	1.30)	296	294	1.37	(1.11,	1.69)				
AG/GG	227	126	0.78	(0.60,	1.01)	210	177	1.16	(0.91,	1.47)	102	122	1.66	(1.24,	2.23)				
*TXNRD2* (rs7410379)																0.0419	0.7961	G = 0 & E = 2	0.0052
GG	396	241	1.00			389	290	1.21	(0.97,	1.52)	198	229	1.87	(1.45,	2.40)				
GA/AA	361	262	1.18	(0.94,	1.48)	406	339	1.35	(1.09,	1.68)	200	187	1.50	(1.16,	1.94)				
*TXNRD3* (rs777238)																0.0301	0.1505	G = 0 & E = 2	0.0015
CC	597	375	1.00			575	454	1.25	(1.04,	1.50)	269	297	1.74	(1.41,	2.15)				
CT/TT	161	129	1.27	(0.97,	1.66)	221	176	1.25	(0.98,	1.58)	129	119	1.41	(1.06,	1.88)				
*SelN1* (rs11247735)																0.0380	0.1536	G = {0,1} & E = 2	<0.0001
GG	219	132	1			219	152	1.13	(0.84,	1.53)	109	124	1.88	(1.34,	2.63)				
GA	369	248	1.11	(0.85,	1.46)	402	323	1.33	(1.02,	1.72)	189	216	1.86	(1.39,	2.49)				
AA	170	124	1.21	(0.88,	1.66)	175	155	1.46	(1.07,	1.99)	100	76	1.23	(0.85,	1.78)				
*SelN1* (rs718391)																0.0025	0.0125	G = {1,2} & E = 2	<0.0001
CC	200	148	1			242	187	1.02	(0.77,	1.37)	127	94	0.97	(0.69,	1.37)				
CG/GG	558	356	0.85	(0.66,	1.10)	554	443	1.06	(0.83,	1.36)	271	322	1.56	(1.20,	2.05)				
**Rectal Cancer**																			
*TXNRD1* (rs17202060)																0.0002	0.0017	G = {1,2} & E = 2	0.192
CC	119	116	1.00			197	125	0.62	(0.44,	0.88)	115	72	0.62	(0.42,	0.92)				
CT	140	99	0.72	(0.50,	1.04)	159	144	0.90	(0.63,	1.27)	94	103	1.08	(0.74,	1.59)				
TT	52	28	0.53	(0.32,	0.91)	51	33	0.63	(0.38,	1.06)	26	29	1.09	(0.60,	1.97)				
*TXNRD1* (rs5018287)																<.0001	0.0005	G = 0 & E = 2	0.3025
GG	101	67	1.00			115	89	1.14	(0.75,	1.73)	55	69	1.85	(1.15,	2.96)				
GA	161	113	1.07	(0.73,	1.59)	196	154	1.16	(0.79,	1.69)	123	105	1.26	(0.84,	1.89)				
AA	49	63	1.91	(1.18,	3.11)	97	59	0.88	(0.56,	1.39)	57	30	0.76	(0.44,	1.30)				

1 Odds Ratios (OR) and 95% Confidence Intervals (CI) adjusted for age, study center, race, and sex.

2 G = numerical coding (i.e., 0, 1, 2) for the SNP; E = numerical coding (i.e., 0, 1, 2) for the environmental factor.

We evaluated these candidate selenoprotein genes with hazard of dying of colorectal cancer after diagnosis with colon or rectal cancer (Table 6). *TXNRD1*, *TXNRD3*, *SeP15*, and *SepX1* were associated with survival after colon cancer diagnosis; *SeP15* and *SepX1* remained significant after FDR multiple comparison adjustment (HRR 1.47, 95% CI 1.13,1.90 and HRR 1.47 95% CI 1.3,1.90 respectively). *TNXRD2*, *SelN1*, and *SepX1* were associated with survival after diagnosis with rectal cancer. *SelN1* rs718391 (HRR 1.67, 95% CI 1.11,2.51) and *SepX1* rs13331553 (HRR 1.46 95%CI 1.07,2.00) and *SepX1* rs732510 (HRR 1.68 95% CI 1.09,2.60) had FDR of <0.10.

**Table 6:** Association between *TNXRD* and Selenoprotein genes and survival after diagnosis with colon and rectal cancer.

Colon Cancer	Death/Person Years	HRR^1^	(95% CI)		Raw *P*	FDR *P*
*TXNRD1* (rs4964778)					0.0407	0.3260
CC	202/5585	1.00				
CG/GG	106/2561	1.28	(1.01,	1.63)		
*TXNRD3* (rs11718498)					0.0301	0.1503
GG/GA	265/6812	1.00				
AA	44/1329	0.70	(0.50,	0.97)		
*SeP15* (rs9433110)					0.0154	0.0461
GG	254/6961	1.00				
GA/AA	55/1187	1.45	(1.07,	1.95)		
*SepX1* (rs732510)					0.0038	0.0076
AA/AG	227/6370	1.00				
GG	81/1729	1.47	(1.13,	1.90)		
**Rectal Cancer**						
*TXNRD2* (rs3788314)					0.0260	0.5042
GG	56/1100	1.00				
GA/AA	115/3190	0.69	(0.49,	0.96)		
*TXNRD2* (rs756661)					0.0504	0.5042
TT/TC	139/3607	1.00				
CC	32/682	1.50	(1.00,	2.24)		
*SelN1* (rs718391)					0.0144	0.0722
CC/CG	137/3482	1.00				
GG	34/807	1.67	(1.11,	2.51)		
*SepX1* (rs13331553)					0.0178	0.0184
TT	78/2155	1.00				
TC/CC	93/2135	1.46	(1.07,	2.00)		
SepX1 (rs732510)					0.0184	0.0184
AA	41/1275	1.00				
AG	80/1974	1.22	(0.83,	1.80)		
GG	49/1022	1.68	(1.09,	2.60)		
P Trend	0.0182					

1 Hazard Rate Ratios (HRR) adjusted for age, study center, race, sex, AJCC stage, and tumor markers.

## Discussion

We observed associations between selenoprotein genes and colon and rectal cancer risk overall as well as from interacting with variables that may influence oxidative stress, including NSAIDs, cigarette smoking, BMI, and estrogen status. However, we observed only minimal interaction with dietary antioxidants, including selenium. In these data *TXNRD1*, *TXNRD2*, *TNXRD3*, *SepX1*, and *SelN1*, and *SeP15* also were associated with survival after diagnosis with colon or rectal cancer. C11orf31 was not associated with colon and rectal cancer through either main or interactive effects.

The thioredoxin system is a major antioxidant system central to intracellular oxidation processes [34], [35], [36]. The major independent associations were observed for *TXNRD1*, *TXNRD2*, *TXNRD3*, and *SelN*. While associations with most SNPs were different for colon and rectal cancer, the same genes appeared to be important. However, *SelN* rs4659382 was associated with significant reduced risk of both colon cancer (OR 0.76) and rectal cancer (OR 0.58). Additionally, multiple SNPs in *SelN* were associated with rectal cancer, as were multiple SNPs in *TXNRD2* for both colon and rectal cancer, although associations did not reach significance after adjustment for multiple comparisons. Others have shown significant associations between *TXNRD1* rs35009941 and colorectal adenomas [37]. Given the extremely rare minor allele frequency of that SNP (only one case of 747 were homozygote variant and four were heterozyote for the variant allele in their study), we did not genotype that SNP. A study by Meplan and colleagues also evaluated several of these genes combining colon and rectal cancers [38]. They observed a significant association with *SelS*, attributing to an inflammation-related pathway; *SelS* has been shown to attenuate inflammation by decreasing pro-inflammatory cytokines [15]. We did not observe an independent association with *SelS*. Hesketh and Meplan have hypothesized that genetic factors could modulate effects at multiple points along a network of pathways [39]. Pathways they cite as potentially important links between selenium, selenoproteins, and colon cancer involve oxidative stress, inflammation, and apoptosis.

Given the hypothesized influence of selenoproteins on oxidative stress and inflammation-related pathways, it is reasonable to determine if factors that alter inflammation such as aspirin/NSAID use and cigarette smoking could modify the risk associated with the genes. We observed that *TNXRD1* and *TNXRD2* interacted with both aspirin and cigarette smoking to alter colon and rectal cancer risk. *TNXRD3* also interacted with aspirin/NSAID use to modify risk of rectal cancer, in that those with the variant genotype who did not use aspirin/NSAID had a similar reduced risk of rectal cancer as those who used aspirin/NSAIDs. These findings suggest that the risk associated with either not using aspirin/NSAID or smoking cigarettes may be influenced by genotype of several selenoprotein genes.

Of interest was the observed interaction between a number of SNPs in selenoprotein genes and estrogen status. Estrogen has anti-inflammatory properties, which could explain some of these associations. However, it also has been shown that estrogen influences tissue distribution and metabolism of selenium [19]. In vitro interaction studies have shown interaction between a splicing variant of *TXNRD1b* and both ERα and ERβ and concluded that it was an important modulator of estrogen signaling [18]. Other selenoproteins could have similar associations with estrogen status. In this study, we observed significant interactions with *TXNRD2*, *SelS*, *SeP15*, and *SelW* with estrogen status, although significance was reduced after multiple comparison adjustment. Although the same SNPs were not associated with colon and rectal cancer, both *TXNRD2* and *SELW* were associated with both tumor sites. Recent estrogen exposure has been associated with reduced risk of colon and rectal cancer; selenoprotein genotypes appear to influence that association.

Of interest was the observation that BMI reacted in a similar manner with *TXNRD1*, *TXNRD2*, and *TXNRD3* as did aspirin/NSAIDs, and smoking cigarettes, and estrogen status. The mechanism underlying these interactions could involve both an inflammation-related pathway and an estrogen-related pathway. The colon and rectal cancer risk associated with BMI was influenced by genotype of these genes. The interaction with BMI was greater for colon cancer than for rectal cancer, however associations with BMI overall appear to influence colon but not rectal cancer [21], [40]. We are unaware of others evaluating the interaction between lifestyle factors and genetic variation in selenoprotein genes. Our results suggest that genetic risk is modified by lifestyle, but confirmation of these findings by others is needed.

Studies have shown that the thioredoxin system can predict prognosis of other types of cancer [34]. *SeP15* has been shown to inhibit tumorigenicity and metastasis of colon cancer cells [20]. In the study by Irons, they observed that *SeP15* influenced expression patterns of over 1000 genes in mice. Those genes that were most commonly influenced were those whose biological function included cellular growth and proliferation. We observed differences in likelihood of dying for several selenoprotein genes, including *SeP15*, which would support the hypothesis that genetic variation in selenoprotein genes may influence survival after diagnosis.

Major strengths of our study were the hypothesis-driven approach, the large and extensive data set that includes information on genetic, diet, and lifestyle data, and our ability to examine colon and rectal cancer separately. While we believe that the data we present are both thorough and informative, we acknowledge that limitations exist. For instance, while we have detected associations we have minimal information on the functionality of the SNPs evaluated. Additional lab-based experiments are needed to determine functionality. Through our analysis we have made many comparisons. We used several methods to adjust for multiple comparisons, the pACT which takes into account the correlated nature of the SNP data, the step-down Holm Bonferroni to adjust for interaction associations, and the maxT which relies on permutation methods. Several interactions were significant after adjusting for multiple comparisons by both methods. The maxT method partitions the data into categories that helps to describe the interaction while the step-down Bonferroni statistic is based on our results from logistic regression models that rely on a common referent point and test for difference in effects across cells of environmental and genetic exposures. We believe that these two methods are complimentary, reinforcing the associations that are significant after multiple testing adjustment and helping to define the elements of the data that are interacting. However, we acknowledge the possibility of chance findings and therefore replication of these results is critical.

Several potential weakness exist. Our study relied on recalled dietary intake to evaluate nutrients such as selenium. Nutrient databases for selenium content of foods can be inaccurate given the selenium content of the soil influences selenium levels in food. Information on source of food could not be obtained in a study such as this given the lack of knowledge of where foods are grown or the selenium content of soil, leaving the possibility of lack of association from misclassification of selenium intake. Unfortunately, given the study design we do not have selenium measurements that would more accurately reflect selenium levels of study participants. Additionally, we have relied on self-reported weight to calculate BMI. We were unable to evaluate change in weight that may be associated. In our study, Hispanic and African American participants had larger mean levels of BMI; however the associations with colon cancer were the same across all ethnic groups.

The study findings support an association between selenoprotein genes and colon and rectal cancer development and survival after diagnosis. Given the interactions observed, it is likely that the impact of cancer susceptibility from genotype is modified by lifestyle factors. The data presented here support the role of selenoproteins in the carcinogenic process and suggest that they may function through pathways that involve inflammation, oxidative stress, and estrogen.

## Supporting Information

Table S1 Associations between dietary variables and selenoprotein genes, adjusted for age, center, race, sex, and kcal.
(DOCX)
