# Supplementary material for: Genetic Variation in Selenoprotein Genes, Lifestyle, and Risk of Colon and Rectal Cancer
Source: PLoS One. 2012 May 17;7(5):e37312. doi: 10.1371/journal.pone.0037312 (PMC3355111; doi:10.1371/journal.pone.0037312)
Supplement: Table S1 — Associations between dietary variables and selenoprotein genes, adjusted for age, center, race, sex, and kcal. (DOCX) [file pone.0037312.s001.docx]

| S1. Associations between dietary variables and selenoprotein genes, adjusted for age, center, race, sex, and kcal. | | | | | | | | | | | | | | | | | |  |
| --- | --- | --- | --- | --- | --- | --- | --- | --- | --- | --- | --- | --- | --- | --- | --- | --- | --- | --- |
|  |  | Low | | | | | Intermediate | | | | | High | | | | |  |  |
|  |  | Controls | Cases | OR | (95% CI) | | Controls | Cases | OR | (95% CI) | | Controls | Cases | OR | (95% CI) | | Raw *P* | Holm's |
| **Colon** | | Vitamin C | | | | | | | | | | | | | | |  |  |
| *TXNRD1* (rs7962759) | | | |  |  |  |  |  |  |  |  |  |  |  |  |  | 0.029 | 0.232 |
|  | CC | 283 | 215 | 1.00 |  |  | 593 | 477 | 1.02 | (0.82, | 1.27) | 294 | 260 | 1.01 | (0.78, | 1.32) |  |  |
|  | CG | 151 | 128 | 1.14 | (0.85, | 1.54) | 296 | 258 | 1.13 | (0.88, | 1.45) | 152 | 103 | 0.77 | (0.56, | 1.06) |  |  |
|  | GG | 24 | 23 | 1.37 | (0.75, | 2.50) | 56 | 30 | 0.72 | (0.44, | 1.16) | 15 | 10 | 0.70 | (0.30, | 1.61) |  |  |
| *TXNRD2* (rs6518591) | | | |  |  |  |  |  |  |  |  |  |  |  |  |  | 0.0347 | 0.694 |
|  | AA | 300 | 253 | 1.00 |  |  | 659 | 508 | 0.88 | (0.72, | 1.08) | 317 | 233 | 0.75 | (0.59, | 0.97) |  |  |
|  | AG | 159 | 113 | 0.84 | (0.62, | 1.13) | 267 | 252 | 1.06 | (0.83, | 1.35) | 146 | 130 | 0.90 | (0.66, | 1.22) |  |  |
|  | GG | 30 | 13 | 0.52 | (0.27, | 1.03) | 55 | 32 | 0.65 | (0.40, | 1.04) | 23 | 20 | 0.83 | (0.44, | 1.57) |  |  |
|  |  | Vitamin E | | | | | | | | | | | | | | |  |  |
| *TXNRD1 (rs7962759)* | | | |  |  |  |  |  |  |  |  |  |  |  |  |  | 0.025 | 0.2 |
|  | CC | 292 | 207 | 1.00 |  |  | 596 | 478 | 1.00 | (0.80, | 1.25) | 282 | 267 | 1.01 | (0.76, | 1.36) |  |  |
|  | CG | 144 | 106 | 1.07 | (0.78, | 1.45) | 291 | 268 | 1.17 | (0.90, | 1.50) | 164 | 115 | 0.76 | (0.54, | 1.07) |  |  |
|  | GG | 22 | 20 | 1.37 | (0.73, | 2.58) | 52 | 31 | 0.79 | (0.49, | 1.29) | 21 | 12 | 0.58 | (0.27, | 1.24) |  |  |
| *TXNRD2 (rs9306229)* | | | |  |  |  |  |  |  |  |  |  |  |  |  |  | 0.0348 | 0.696 |
|  | CC | 280 | 216 | 1.00 |  |  | 598 | 480 | 0.92 | (0.73, | 1.15) | 298 | 228 | 0.74 | (0.55, | 1.01) |  |  |
|  | CT | 175 | 117 | 0.87 | (0.65, | 1.17) | 329 | 262 | 0.92 | (0.72, | 1.19) | 153 | 154 | 1.03 | (0.74, | 1.42) |  |  |
|  | TT | 32 | 14 | 0.57 | (0.30, | 1.10) | 55 | 57 | 1.21 | (0.80, | 1.84) | 34 | 25 | 0.74 | (0.42, | 1.31) |  |  |
| *TXNRD3 (rs11718498)* | | | |  |  |  |  |  |  |  |  |  |  |  |  |  | 0.0049 | 0.0245 |
|  | GG/GA | 380 | 295 | 1.00 |  |  | 814 | 684 | 0.96 | (0.79, | 1.17) | 407 | 327 | 0.78 | (0.60, | 1.02) |  |  |
|  | AA | 107 | 53 | 0.65 | (0.45, | 0.93) | 170 | 115 | 0.77 | (0.58, | 1.04) | 78 | 80 | 1.04 | (0.71, | 1.52) |  |  |
|  |  | Beta Carotene | | | | | | | | | | | | | | |  |  |
| *TXNRD2 (rs1978058)* | | | |  |  |  |  |  |  |  |  |  |  |  |  |  | 0.0076 | 0.152 |
|  | CC | 192 | 138 | 1.00 |  |  | 391 | 320 | 1.06 | (0.81, | 1.38) | 179 | 158 | 1.05 | (0.77, | 1.44) |  |  |
|  | CT | 231 | 178 | 1.08 | (0.80, | 1.44) | 436 | 351 | 1.05 | (0.81, | 1.37) | 220 | 183 | 0.97 | (0.72, | 1.32) |  |  |
|  | TT | 66 | 68 | 1.46 | (0.98, | 2.20) | 153 | 112 | 0.96 | (0.69, | 1.33) | 84 | 46 | 0.64 | (0.41, | 0.98) |  |  |
| *TXNRD3 (rs777226)* | | | |  |  |  |  |  |  |  |  |  |  |  |  |  | 0.002 | 0.01 |
|  | GG/GA | 466 | 373 | 1.00 |  |  | 931 | 753 | 0.94 | (0.79, | 1.11) | 471 | 363 | 0.81 | (0.65, | 0.99) |  |  |
|  | AA | 24 | 11 | 0.55 | (0.26, | 1.13) | 51 | 31 | 0.70 | (0.44, | 1.12) | 13 | 24 | 2.00 | (1.00, | 4.01) |  |  |
|  |  | Selenium | | | | | | | | | | | | | | |  |  |
| *TXNRD1 (rs7962759)* | | | |  |  |  |  |  |  |  |  |  |  |  |  |  | 0.013 | 0.104 |
|  | CC | 291 | 194 | 1.00 |  |  | 603 | 496 | 1.14 | (0.91, | 1.44) | 276 | 262 | 1.12 | (0.82, | 1.54) |  |  |
|  | CG | 143 | 114 | 1.25 | (0.92, | 1.70) | 285 | 248 | 1.23 | (0.95, | 1.60) | 171 | 127 | 0.88 | (0.62, | 1.26) |  |  |
|  | GG | 25 | 20 | 1.31 | (0.71, | 2.44) | 47 | 30 | 0.92 | (0.56, | 1.52) | 23 | 13 | 0.67 | (0.32, | 1.39) |  |  |
| *TXNRD2 (rs17745445)* | | | |  |  |  |  |  |  |  |  |  |  |  |  |  | 0.0408 | 0.7344 |
|  | GG | 367 | 249 | 1.00 |  |  | 721 | 578 | 1.09 | (0.89, | 1.34) | 358 | 319 | 1.04 | (0.78, | 1.39) |  |  |
|  | GA | 116 | 79 | 1.02 | (0.73, | 1.41) | 231 | 205 | 1.20 | (0.92, | 1.55) | 121 | 89 | 0.83 | (0.57, | 1.22) |  |  |
|  | AA | 6 | 11 | 2.70 | (0.98, | 7.42) | 23 | 19 | 1.14 | (0.61, | 2.16) | 12 | 6 | 0.55 | (0.20, | 1.51) |  |  |
| *TXNRD2 (rs5992493)* | | | |  |  |  |  |  |  |  |  |  |  |  |  |  | 0.0269 | 0.5111 |
|  | AA/AG | 478 | 316 | 1.00 |  |  | 935 | 764 | 1.14 | (0.94, | 1.37) | 475 | 400 | 1.00 | (0.76, | 1.32) |  |  |
|  | GG | 11 | 23 | 3.04 | (1.45, | 6.36) | 40 | 38 | 1.31 | (0.82, | 2.11) | 16 | 14 | 0.96 | (0.45, | 2.07) |  |  |
| *TXNRD2 (rs6518591)* | | | |  |  |  |  |  |  |  |  |  |  |  |  |  | 0.0033 | 0.066 |
|  | AA | 308 | 229 | 1.00 |  |  | 637 | 518 | 1.00 | (0.80, | 1.25) | 331 | 247 | 0.79 | (0.58, | 1.07) |  |  |
|  | AG | 151 | 101 | 0.88 | (0.65, | 1.19) | 285 | 252 | 1.08 | (0.84, | 1.39) | 136 | 142 | 1.09 | (0.76, | 1.55) |  |  |
|  | GG | 30 | 9 | 0.40 | (0.18, | 0.85) | 54 | 32 | 0.71 | (0.44, | 1.15) | 24 | 24 | 1.03 | (0.55, | 1.94) |  |  |
|  |  |  |  |  |  |  |  |  |  |  |  |  |  |  |  |  |  |  |
| **Rectal** | | Vitamin C | | | | | | | | | | | | | | |  |  |
| *TXNRD2 (rs6518591)* | | | |  |  |  |  |  |  |  |  |  |  |  |  |  | 0.0404 | 0.808 |
|  | AA | 174 | 142 | 1.00 |  |  | 318 | 229 | 0.81 | (0.61, | 1.08) | 142 | 135 | 0.95 | (0.67, | 1.36) |  |  |
|  | AG | 63 | 64 | 1.24 | (0.82, | 1.88) | 150 | 113 | 0.87 | (0.62, | 1.21) | 87 | 53 | 0.60 | (0.39, | 0.93) |  |  |
|  | GG | 5 | 4 | 0.87 | (0.23, | 3.33) | 10 | 9 | 1.06 | (0.42, | 2.69) | 9 | 5 | 0.57 | (0.18, | 1.76) |  |  |
|  |  | Beta Carotene | | | | | | | | | | | | | | |  |  |
| *TXNRD2 (rs3788305)* | | | |  |  |  |  |  |  |  |  |  |  |  |  |  | 0.0237 | 0.474 |
|  | AA | 63 | 74 | 1.00 |  |  | 138 | 104 | 0.57 | (0.37, | 0.88) | 74 | 45 | 0.40 | (0.24, | 0.68) |  |  |
|  | AG | 110 | 89 | 0.69 | (0.44, | 1.06) | 215 | 188 | 0.67 | (0.45, | 1.00) | 105 | 77 | 0.52 | (0.32, | 0.82) |  |  |
|  | GG | 68 | 49 | 0.58 | (0.35, | 0.96) | 128 | 78 | 0.46 | (0.29, | 0.71) | 58 | 50 | 0.53 | (0.31, | 0.91) |  |  |
| *TXNRD2 (rs3788306)* | | | |  |  |  |  |  |  |  |  |  |  |  |  |  | 0.0266 | 0.5054 |
|  | TT | 118 | 118 | 1.00 |  |  | 239 | 177 | 0.67 | (0.48, | 0.92) | 129 | 82 | 0.50 | (0.33, | 0.75) |  |  |
|  | TC | 92 | 74 | 0.80 | (0.54, | 1.20) | 203 | 155 | 0.70 | (0.50, | 0.98) | 88 | 72 | 0.66 | (0.43, | 1.01) |  |  |
|  | CC | 31 | 20 | 0.62 | (0.33, | 1.15) | 39 | 38 | 0.88 | (0.52, | 1.49) | 20 | 18 | 0.71 | (0.35, | 1.44) |  |  |
| *TXNRD2 (rs9605030)* | | | |  |  |  |  |  |  |  |  |  |  |  |  |  | 0.0384 | 0.6912 |
|  | CC | 167 | 160 | 1.00 |  |  | 353 | 279 | 0.75 | (0.57, | 0.99) | 173 | 114 | 0.55 | (0.39, | 0.78) |  |  |
|  | CT/TT | 74 | 52 | 0.73 | (0.48, | 1.11) | 127 | 91 | 0.69 | (0.48, | 0.98) | 64 | 58 | 0.76 | (0.49, | 1.18) |  |  |
|  |  |  |  |  |  |  |  |  |  |  |  |  |  |  |  |  |  |  |
